# Race in Integrated Data Systems: Why, Wherefrom, and How

**DOI:** 10.23889/ijpds.v9i5.2623

**Published:** 2024-09-18

**Authors:** Stephen Steh, Francisca Richter, Robert Fischer, Meagan Ray-Novak, Michael Henderson

**Affiliations:** 1 Case Western Reserve University

Integrated administrative data systems (IADs) are a powerful resource to implement research and analysis for social policy. While IADs may capture racial identity data from multiple administrative sources, there is no agreed upon criteria for whether and how to synthesize this information in a way that (1) produces knowledge to advance racial equity, while (2) underscoring race as a social construct. This study leverages a county-level IAD to test and analyze an event-table design to represent racial identity that, when informed with historical and community knowledge, may meet both of the aforementioned goals.

We illustrate this approach applied to the development of a registry of youth experiencing homelessness, as captured by linked administrative data from vital records, homeless service agencies, schools, food support programs, and other systems. The event table design includes race identity for each person in the registry across all systems included. We develop criteria to inform the hierarchy of one source over another when there are discrepancies in race identity across systems, and when categories for this variable differ across administrative systems. We provide historical and social context behind potential discrepancies and discuss approaches to missing data. Furthermore, we highlight the value of including qualitative knowledge from agency data managers, users, and those represented in the data to inform the synthesis of information around race. Finally, we illustrate how this approach can guide research analysis and contextualize results, thus enhancing the research process and advancing racial equity with IADs.

